# The impact of the Covid-19 pandemic on quality of life in skin cancer patients

**DOI:** 10.1371/journal.pone.0255501

**Published:** 2021-08-18

**Authors:** Jonas K. Kurzhals, Gina Klee, Hauke Busch, Victoria Hagelstein, Detlef Zillikens, Patrick Terheyden, Ewan A. Langan

**Affiliations:** 1 Department of Dermatology, University of Lübeck, Lübeck, Germany; 2 Lübeck Institute of Experimental Dermatology, University of Lübeck, Lübeck, Germany; 3 Dermatological Sciences, University of Manchester, Manchester, England, United Kingdom; The University of Sydney, AUSTRALIA

## Abstract

With more than 82 million cases worldwide and almost two million deaths, the Covid-19 global pandemic shows little sign of abating. However, its effect on quality of life (QoL) in skin cancer patients has not been systematically evaluated to date. Given that QoL impairments may be associated with increased psychological morbidity, and may interfere with engagement with cancer therapy and follow-up, we prospectively evaluated quality of life in skin cancer patients using the Covid-19 Emotional Impact Survey (C-19EIS) and the EORTC QLQ-C30 questionnaires. 101 patients (48 females and 53 males) completed both questionnaires. The mean C-19EIS score was 3.8 on a scale from 0 (no impact) to 12 (severe impact). Patients undergoing systemic therapy showed significantly impaired physical (p = 0.006) and social functioning (p = 0.003). However, when compared to the published normative EORTC QLQ-C30 data, there was no evidence that the Covid-19 pandemic had significantly impacted upon overall quality of life. Subscales of the EORTC QLQ-C30 were significantly inversely correlated with the C-19EIS, validating its use in skin cancer patients. Despite the Covid-19 pandemic, skin cancer patients in our tertiary referral center were surprisingly resilient. However, given the geographical variations in the rates of Sars-CoV-2 infection it is possible that the low incidence in Northern Germany may have resulted in a lack of general QoL impairments. Multi-center studies are required to further determine the impact of Covid-19 on psychological wellbeing in skin cancer patients in order to develop supportive interventions and to ensure that engagement with cancer care services is maintained in order to enable early detection of cancer progression and/or recurrence.

## Introduction

The SARS-CoV-2 virus is a member of the coronavirus family and is responsible for the development of Covid-19 disease. Initially considered to be primarily a pulmonary disease, it is now clear that Covid-19 is a multi-system infection that can be associated with significant morbidity and mortality. With more than 82 million cases of SARS-CoV-2 infection worldwide since the beginning of the pandemic, Covid-19 represents a major and ongoing global healthcare challenge [1] with a dramatic impact on employment, education, and psychological wellbeing. The impact of treating patients with Covid-19 on healthcare systems, coupled with its high rates of transmission [2], has resulted in an unprecedented race to develop an effective vaccine. Indeed, two RNA-based vaccines have now been licensed for both use in the United States and Europe, with other vaccines expected to receive regulatory approval in the near future [3–5].

This first wave of the COVID-19 pandemic was associated with a significant impact upon psychosocial wellbeing [6]. Whilst the development of vaccines promises to dramatically alter the course of the pandemic in the medium to long term and may reassure patients, especially those in risk groups, that the disease can be brought under control [3, 5] little is known about the specific impact of Covid-19 on the quality of life in patients with skin cancer, especially in the context of the second wave in Europe. This is perhaps surprising given the incidence and prevalence of skin cancer and that patients with advanced skin cancer have to attend healthcare facilities for diagnostic procedures, treatment (including surgical intervention, radio- and or chemo/immunotherapy), and follow-up/prevention appointments.

Patients suffering from cancer are clearly a vulnerable group in the Covid-19 pandemic. Due to an immunocompromised/-suppressed status and dependent on the underlying tumor disease and burden, cancer patients may be at an increased risk of developing severe Covid-19 disease and requiring treatment in an intensive care setting [7]. Kuderer et al. illustrated that patients with an ECOG performance status of more than 2 are at higher risk of suffering a poorer outcome from Covid-19 [8]. Ciążyńska et al. reported that Covid-19 significant impacts upon cancer patients’ quality of life [9]. In terms of skin cancer patients in particular, the diagnosis of melanoma and its treatment are both associated with significant impairments in quality of life and increased levels of distress [10].

However, the advent of immunotherapy has dramatically improved both overall and progression-free survival for patients suffering from melanoma and other skin cancers [11–13]. The risk and course of infection with Sars-CoV-2 in patients undergoing immunotherapy for advanced and/or metastatic skin cancer are still being evaluated [14]. Given the lack of data on the impact of Covid-19 on quality of life in patients with skin cancer, we utilized the Covid-19 Emotional Impact Survey (C-19EIS) and EORTC Q30 to determine whether the quality of life changes were determinable and whether these were restricted to patients undergoing systemic treatment (immunotherapy or targeted therapy). We postulated that the spiraling levels of infection provided the ideal time point at which to capture any impact on the quality of life.

## Methods

In order to provide a robust assessment of the impact of Covid-19 on quality of life two questionnaires were used. Both were offered to patients to complete on a voluntary basis after written informed consent was obtained according to the Declaration of Helsinki principles. Ethical approval was obtained from the University of Lübeck ethics committee (Reference number: 20–363). The questionnaires were distributed and completed between the 1^st^ and 30^th^ of November 2020 which coincided with the second national lockdown (lockdown-light) in Germany, a period marked by a dramatic increase in the number of daily new infections. All patients were recruited from the dermato-oncology outpatient setting, either attending for routine follow-up (group 1) or systemic therapy (group 2). The systemic therapies were primarily immunotherapy (immune-checkpoint inhibition) but also included patients undergoing targeted (BRAF and MEK inhibition) or chemotherapy (dacarbazine). The sample size reflected the number of skin cancer patients attending the outpatient clinic or immunotherapy unit in November 2020 who were willing to participate in our study. Only patients aged over 18 years were eligible to participate.

Given that no specific questionnaire has been developed to determine the impact of COVID-19 on quality of life in patients with skin cancer, we used the Covid-19 Emotional Impact Survey (C-19EIS) developed by Falcone et al. 2020. Not only was the C-19EIS developed in Italy, a country severely affected during the first wave of the pandemic, but was also designed for use in patients with cancer [15]. Moreover, the C-19EIS had been validated in previous publications [15] and found to reliably and robustly capture changes in quality of life which were stable over time. In brief, the C-19EIS includes a 6-item-core component (Table 1). The score ranges from 0 (not impact) to 12 (severe impact). Permission to use the Covid-19 impact survey was obtained from Falcone et al (personal communication). Age was recorded given that it is a significant risk factor for Covid-19 related mortality. In addition, the following co-morbidities were recorded; smoking status, anticoagulation therapy, arterial hypertension, coronary heart disease, and diabetes mellitus, representing additional risk factors for severe Covid-19 disease [16].

**Table 1:** The six core components of the Covid-19 Emotional Impact Survey (C-19EIS).

1. Are you experiencing fear / anxiety related to the Covid-19 pandemic?	yes/no
2. Has the onset of the Covid-19 outbreak left you feeling less medically protected?	yes/no
3. Do you believe your disease will be affected by the Covid-19 outbreak?	yes/no
4. Has the Covid-19 outbreak changed how you perceive your disease?	yes/no
5. How much impact is the Covid-19 outbreak having on the quality of your life?	0; 1; 2; 3; 4
6. How much impact is the Covid-19 outbreak having on your emotional state?	0; 1; 2; 3; 4

In addition to the Covid-19 impact survey, patients were asked to complete the EORTC QLQ-C30 questionnaire. This is a well-validated and recognized questionnaire to measure the quality of life in patients with cancer [17, 18] It consists of 30 items, forming different subscales: The global health/quality of life subscale, five functional scales (physical, role, emotional, cognitive, and social), and nine symptom subscales. Subscale scores were calculated as described in the EORTC QLQ-C30 scoring manual (range: from 0–100) and compared with the published normative data [19]. A high score for global health status and the functional subscales represent a high quality of life or a high level of functioning. In contrast, a high score for a symptom scale described more severe symptomatology, for example nausea and fatigue. Permission to use the EORTC questionnaire was obtained before commencing the study (EORTC-questionnaire Request ID:70776).

### Statistical methods

The Spearman rank correlation was used to initially determine whether there was a significant correlation between responses to the Covid-19 Emotional Impact Survey and the QLQ-C 30. Both parametric and non-parametric tests were used. Analyses were then performed using the unpaired t-test or Man-Whitney Test depending on the normal distribution and variances. All data were analyzed using GraphPad Prism version 8, Microsoft Excel (2017), and the R software package. P values < 0.05 are were considered significant.

## Results

### Patients with skin cancer reported low levels of concern regarding the impact of Covid-19 on their quality of life

**Fig 1:**
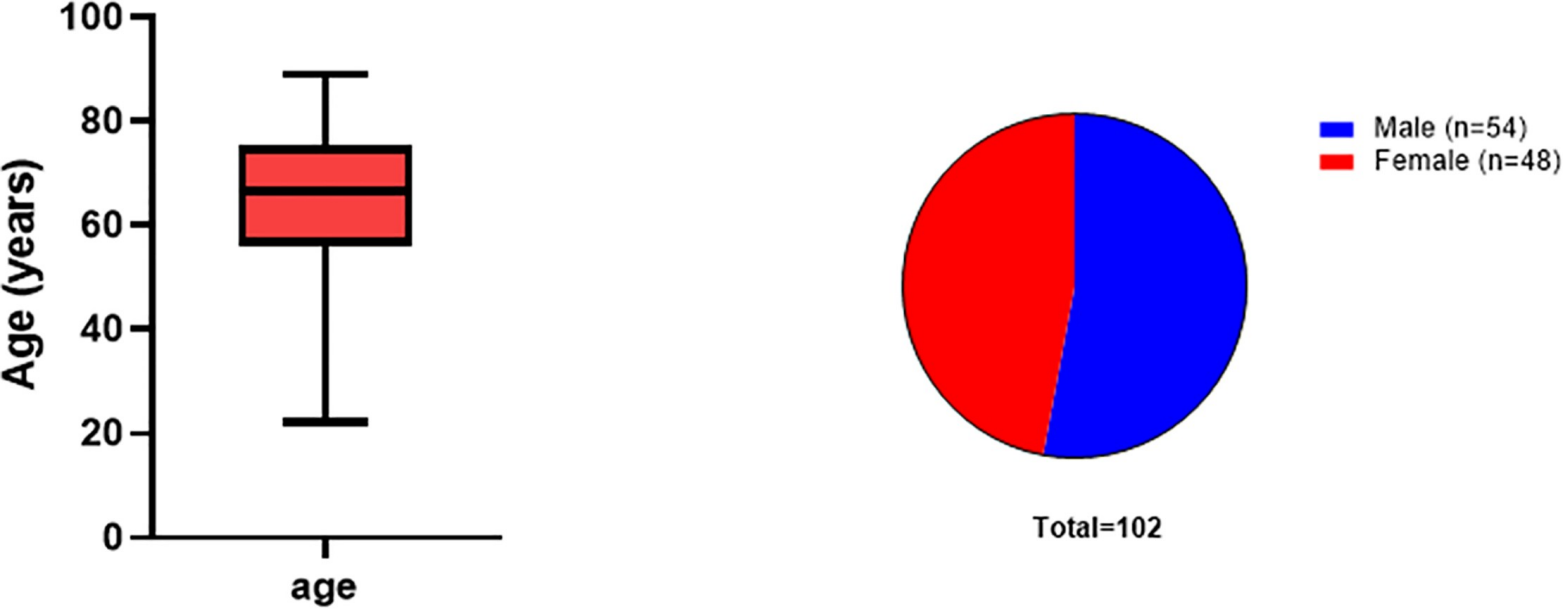
Patient demographics including age (A) and sex (B).

**Fig 2:**
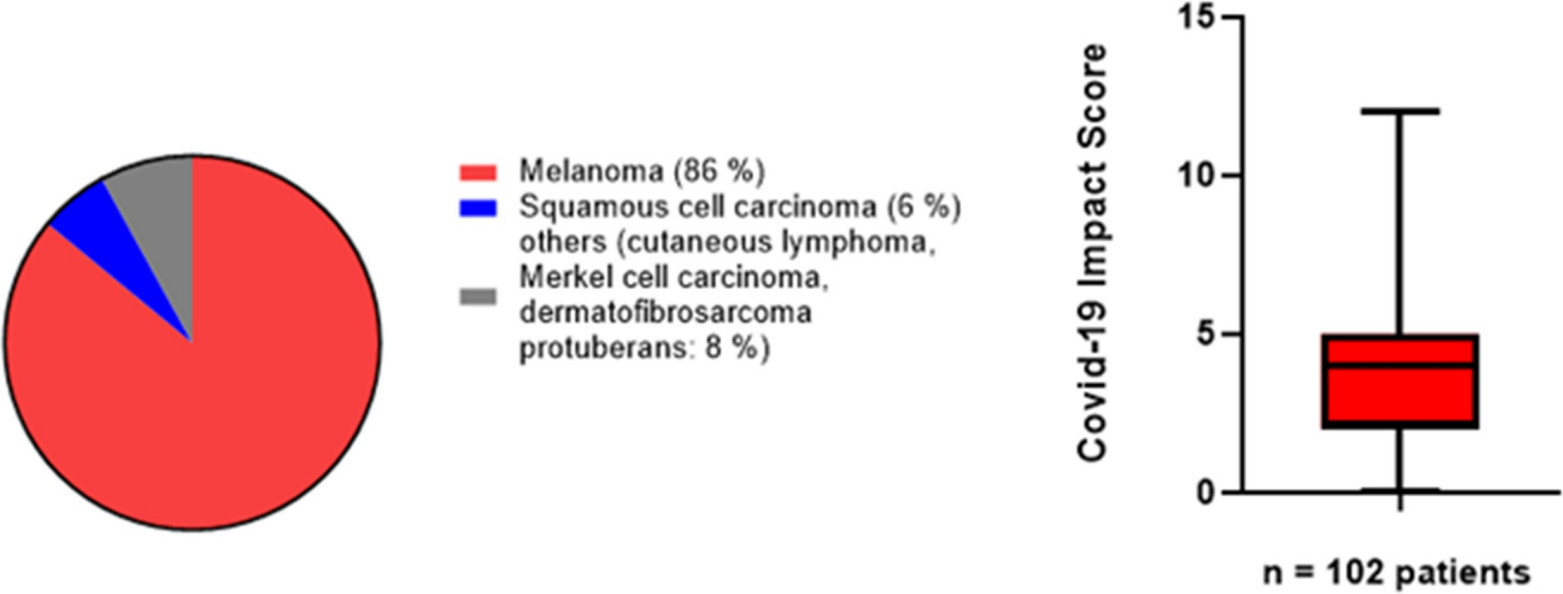
Distribution of the different types of skin cancer (A) and Covid-19 Impact Score (B).

A total of 107 patients completed the questionnaires during the study period (1^st^ until the 30^th^ of November). Incomplete questionnaires were excluded from further analysis. For the final analysis, 102 fully completed C-19EIS questionnaires and 101 QLQ-C 30 were evaluated. The mean age of our patients was 65 +/-14.4 years (range:22–89 years, consisting of 54 males and 48 females (Fig 1). The majority of the patients had melanoma (86%), followed by squamous cell carcinoma a (6%) and others (cutaneous lymphoma, Merkel cell carcinoma, dermatofibrosarcoma protuberans) (8%) (**Fig 2**). The overall mean score for the Covid-19 impact survey was 3.8 **+/-2.2** (range: 0–12). There was no significant difference between the scores of patients in group 1 and 2 (p = 0.3). When the results were analyzed according to the presence of risk factors for severe Covid-19 [20], there were also no significant differences in Covid-19 impact scores between the groups (Table 2).

**Table 2:** Results of the Covid-19 Emotional Impact Survey (C-19EIS).

Factor	All Patients	Group 1	Group 2	p-value
Age	65	63	67	
C19 EIS	3.8	3.7	4.1	n.s
Male	54	32	22	
C19 EIS	3.6	3.5	3.9	n.s
Female	48	55	13	
C19 EIS	4.1	4	4.5	n.s
Diabetes mellitus	6	3	3	
C19 EIS	3.8	4	3.7	n.s
Hypertension	29	18	11	
C19 EIS	4	3.7	4.3	n.s
Cancer type				
Melanoma	88	56	32	
C19 EIS	3.8	3.6	4	n.s

### Global health status was significantly better in skin cancer versus “all cancer” patients despite the Covid-19 pandemic

Next, we analyzed the results of the EORTC QLQ-C30 questionnaires and compared the results to the published normative data for patients with cancer (n = 23553) [21] and then healthy controls [19]. Surprisingly, patients with skin cancer actually reported overall quality of life scores which were significantly higher than patients with all types of cancer (p = 0.02). In emotional (p = 0.009) and social functioning (p = 0.02), our cohort fared significantly better than “all cancer” patients despite the Covid-19 pandemic (Table 3). Finally, when considering the symptom scales, patients with skin cancer reported significantly less symptoms than cancer patients in general (Table 3). Even financial impact scores were significantly lower in our cohort (p = 0.003).

**Table 3:** EORTC quality of life questionnaire—C30 in comparison to reference values "all cancers/all stages".

	During the Covid 19-pandemic		Reference values (all cancer patients during "normal" conditions)		
	n = 101		n = 23553		
	Mean	Standard derivation	Mean	Standard derivation	P value
Global health status/QoL	67.4	23.2	61.3	24.2	p = 0.02
Physical functioning	80	22.8	76.7	23.2	p = 0.13
Role functioning	74.5	27.4	70.5	32.8	p = 0.22
Emotional functioning	77.7	23.6	71.4	24.2	p = 0.009
Cognitive functioning	86.2	22.1	82.6	21.9	p = 0.12
Social functioning	81.3	24.6	75	29	p = 0.02
Fatigue	26.1	27.2	34.6	27.8	p = 0.0022
Nausea and vomiting	4	12.1	9.1	19	p = 0.0070
Pain	19.3	28.1	26.4	30.2	p = 0.0101
Dyspnea	19.7	27.7	21	28.4	p = 0.4303
Insomnia	25	34	28.9	31.9	p = 0.2203
Appetite loss	10	21	21.1	31.3	p = 0.0004
Constipation	10.3	24	17.5	28.4	p = 0.0113
Diarrhea	6.7	17.1	9	20.3	p = 0.2355
*Financial difficulties*	8	22.3	16.3	28.1	p = 0.003

Since there is no published specific reference values for skin cancer in general, we additionally compared our 101 patients to the reference group of “melanoma at all stages” of the EORTC reference values, bearing in mind that almost 90 percent of the patients who completed the questionnaire were suffering from malignant melanoma (Table 4). Again, our patients performed significantly better in terms of emotional functioning (p = 0.047), but there were no differences in the global health status and social functioning. Only dyspnoea was a more common symptom in our cohort.

**Table 4:** EORTC quality of life questionnaire—C30 in comparison to reference values "melanoma/all stages".

	During the Covid 19-pandemic		Reference values (melanoma all stages)		
	n = 101		n = 1200		
	Mean	Standard derivation	Mean	Standard derivation	P value
Global health status/QoL	67.4	23.2	68.2	21	p = 0.7
Physical functioning	80	22.8	*no reference data available*		
Role functioning	74.5	27.4	71.2	32.2	p = 0.3
Emotional functioning	77.7	23.6	73.1	22.3	p = 0.047
Cognitive functioning	86.2	22.1	88.3	17.8	p = 0.26
Social functioning	81.3	24.6	79	26.2	p = 0.39
Fatigue	26.1	27.2	27	25	p = 0.7
Nausea and vomiting	4	12.1	5.6	14.4	p = 0.67
Pain	19.3	28.1	20.7	25.8	p = 0.6
Dyspnoea	19.7	27.7	11	21.4	p = 0.0001
Insomnia	25	34	25	29.9	p = 0.9
Appetite loss	10	21	13.2	25.2	p = 0.21
Constipation	10.3	24	9.5	21.6	p = 0.72
Diarrhoea	6.7	17.1	5.5	15.6	p = 0.46
*Financial difficulties*	8	22.3	15.3	26.9	p = 0.0081

We also compared our cohort to the normative data for the general population (healthy individuals, n = 15386) (Table 5) [19] Skin cancer patients had a similar global health status to healthy controls (p = 0.55). However, physical, role, and social functioning were all significantly impaired in patients with skin cancer (Table 5). Reassuringly, there were no significant differences in any of the symptom scales between our cohort and healthy controls.

**Table 5:** EORTC quality of life questionnaire—C30 in comparison to healthy normal individuals.

	During Covid—19 pandemic		Reference values (Normal data of healthy individuals from 15 countries)		
	n = 101		n = 15386		
	Mean	Standard derivation	Mean	Standard derivation	P value
Global health status/QoL	67.4	23.2	66.1	21.7	p = 0.5435
Physical functioning	80	22.8	85.1	18.9	p = 0.0069
Role functioning	74.5	27.4	84.3	24.6	p = 0.0001
Emotional functioning	77.7	23.6	74.2	24.7	p = 0.1597
Cognitive functioning	86.2	22.1	84.8	21.3	p = 0.5205
Social functioning	81.3	24.6	86.2	24.1	p = 0.04
Fatigue	26.1	27.2	29.5	25.5	p = 0.1833
Nausea and vomiting	4	12.1	5.9	16	p = 0.2336
Pain	19.3	28.1	23.5	27.1	p = 0.1
Dyspnoea	19.7	27.7	15.9	24.6	p = 0.1
Insomnia	25	34	26.6	30.3	p = 0.7412
Appetite loss	10	21	10	21.6	p = 1
Constipation	10.3	24	12.5	23.3	p = 0.3517
Diarrhoea	6.7	17.1	9.5	20.9	p = 0.174
*Financial difficulties*	8	22.3	10.6	23.6	p = 0.2696

To determine whether differences in quality of life were associated with treatment, we further analyzed the EORTC data according to treatment group (Table 6). Again, there was no significant difference between the groups in terms of global health. As expected, physical and social functioning was significantly impaired in patients in group 2 when compared to that in group 1 (p = 0.006 and p = 0.003 respectively). As expected, patients undergoing outpatient therapy for locally advanced and/or metastatic skin cancer reported significantly more symptoms, namely fatigue (p = 0.002), dyspnoea (p = 0.009), and constipation (p = 0.01).

**Table 6:** EORTC quality of life questionnaire—C30 of both our groups.

	Group 1		Group 2		
	n = 66		n = 35		
	Mean	Standard derivation	Mean	Standard derivation	P value
Global health status/QoL	70	24.02	62.62	21.04	p = 0.06
Physical functioning	83.9	21.3	72.7	21.9	p = 0.0056
Role functioning	77.7	26.7	68.57	27.9	p = 0.1
Emotional functioning	80.1	22.2	73.1	25.57	p = 0.15
Cognitive functioning	88.9	19.6	80.95	25.61	p = 0.09
Social functioning	85.6	23.7	73.33	24.65	p = 0.0033
Fatigue	20.5	26.6	36.51	25.5	p = 0.0020
Nausea and vomiting	2.5	8.9	6.667	16.27	p = 0.15
Pain	17.1	28.1	23.33	28.06	p = 0.1
Dyspnoea	14.8	25.7	28.57	29.31	p = 0.0093
Insomnia	21	32.5	32.38	35.69	p = 0.0846
Appetite loss	5.6	16.2	18.1	26	p = 0.0017
Constipation	6.1	18.5	18.1	30.62	p = 0.0096
Diarrohea	6.6	15.8	6.667	19.47	p = 0.7
*Financial difficulties*	5.1	15.8	13.33	30.46	p = 0.25

**Fig 3:**
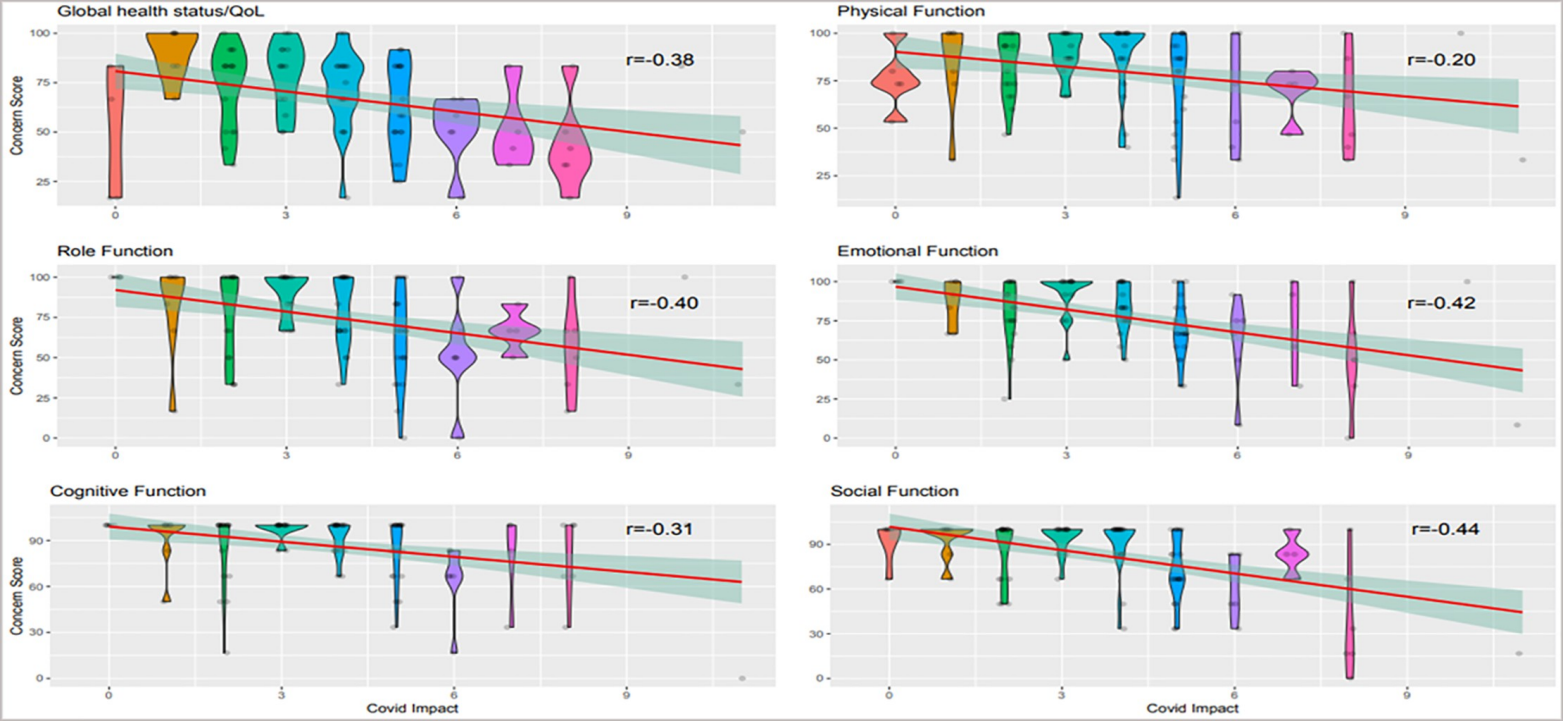
The violin/scatter plots depict the concern scores as a function of the Covid Impact for six health categories. The red line is a linear fit to the data alongside the 95% confidence intervals shaded in green. The numbers show the Spearman’s rho coefficient. All correlations are significantly negative (p< 0.05).

Finally, to validate the Covid-19 Emotional Impact Survey in patients with skin cancer we compared the results with those of the EORTC QLQ-C30 using spearman´s rho correlation coefficient (Fig 3). Covid-19 Emotional Impact Survey Score was significantly inversely correlated with Global health/QoL scale (rho: - 0.38, p = 0.0001), the physical function scale (rho:-0.20, p = 0.0428), the role functioning scale (rho:-0.40, p<0.0001), the emotional functioning scale (rho:-0.42, p<0.0001), the cognitive function scale (rho:-0.31, p = 0. 0016) and the social function scale (rho:-0.44, p<0.0001), largely consistent with Falcone et. al [15].

## Discussion

The Covid-19 pandemic continues to affect physical and mental health on a global and unprecedented scale [22, 23]. Despite the development of several effective vaccines, the virus will continue to significantly impair physical health and mental wellbeing until high levels of immunity are achieved. Vulnerable patient groups, for example, patients suffering from cancer, may not only experience increased anxiety related to the possibility of infection and increased mortality [24] but may also suffer from the effect of measures introduced to ensure their safety, including reduced social contact, social distancing and shielding. Indeed, there is evidence that the Covid-19 pandemic, particularly the first wave, significantly impaired emotional wellbeing in patients with cancer in general [9, 15]. However, little attention has been paid to date on the impact of the Covid-19 pandemic on the quality of life and emotional wellbeing of patients with skin cancer, despite the fact that skin cancer is the fifth most common type of cancer worldwide (https://www.who.int/news-room/fact-sheets/detail/cancer).

Therefore, we sought to ascertain and chart the impact of the Covid-19 pandemic on patients with skin cancer in a tertiary referral center during the second nationwide lockdown in Germany. To our surprise, the overall C-19EIS score was low, at 3.8. Moreover, there was no significant difference in the Covid-19 quality of life impact score between patients who were attending routine out-patient skin cancer follow-up appointments and those attending for systemic anti-cancer therapy. The C-19EIS was developed and validated by Falcone et al. [15] in Italy in patients with thyroid cancer during the first wave of the Covid-19 pandemic. In that patient population, at that time, the average impact score was over 2-fold higher than in our cohort during the second wave of the pandemic. There are a number of possible reasons why the C-19EIS score was lower in our cohort. Perhaps most importantly, Italy was particularly badly affected during the first wave of the pandemic [25], and the health care system struggled to cope with the number of patients requiring intensive care treatment. To date, this situation has not been widely replicated in Germany, although several regions are now also reaching their intensive care capacity. At the time of writing, only 75 patients with Covid-19 are being treated in intensive care in Schleswig Holstein, with 47 requiring mechanical ventilation [26] This may have resulted in reduced levels of concern in our cohort. Moreover, the impact of the development of effective vaccines may also have reduced anxiety in our patients. It is also conceivable that patients had become more accustomed to life under the Covid-19 pandemic and that this contributed to reduced levels of anxiety. It is also possible that patients who were most anxious had already cancelled their treatment or follow-up appointments, although only 2 patients cancelled their appointments due to concerns over the risk of Sars-CoV-2 infection.

Surprisingly, our patients scored significantly better on the EORTC QLQ-C30 measure of global health when compared to cancer patients in general. In fact, their levels of global health were similar to those in the general population. Whilst this is reassuring, it should be borne in mind that the normative data for the EORTC QLQ-C30 for both cancer patients and healthy individuals was not obtained during a global pandemic but is historic in nature. In addition, patients scored also significantly better on the measure of social functioning and emotional functioning in comparison to all cancers at all stages and the improved emotional functioning was maintained when only patients with melanoma were compared (Table 4). The high scores for emotional functioning may reflect also our extensive psycho-oncological support infrastructure, which includes psychologists, social workers, and palliative specialists.

No details were obtained regarding the patients’ direct experience with Covid-19. It is conceivable that patients who had recovered from Sars-CoV-2 infection may have been less anxious. On the other hand, patients who had direct experience with friends or family members who were seriously ill with, or had died due to, Covid-19 infection may have expressed an increased impact on quality of life. This did not seem to be the case in our cohort given that there we no significant overall quality of life impairments.

Moreover, although melanoma is a highly aggressive and life-threatening tumor, depending on the stage of the disease and melanoma subtype [27] we may have detected cancer-type specific differences in quality of life. This finding is supported by evidence that under non-pandemic conditions, patients with melanoma (all stages) have a significantly higher global health status (p = 0.001) as well as superior social functioning (p = 0,0001) and emotional functioning (p = 0.0172) scores than “all cancer” patients (Table 4) [21]. Given that the vast majority of patients in our cohort suffered from melanoma (almost 90%), larger studies are required to determine whether quality of life changes are dependent on skin cancer type.

Nevertheless, physical, role and social functioning were impaired in our cohort compared to the healthy population. Furthermore, scores of physical and social function were significantly worse in the patients attending routine skin cancer follow-up care than in those attending for systemic therapy. It is likely that the impact on social functioning reflected the national recommendations on social distancing and dramatically reducing social contact. This can only be confirmed by repeating the questionnaire when the “lockdown” recommendations have been eased.

Finally, we were able to essentially replicate the results of Falcone et. al [15] that the Covid-19 Emotional Impact Survey score was significantly inversely correlated with the respective sections of the EORTC QLQ-C30 questionnaire, confirming its suitability for the use to determine the effect of the Covid-19 pandemic on the wellbeing of skin cancer patients.

In summary, in one of the first comprehensive assessments of the effect of the Covid-19 pandemic on patients with skin cancer, we were able to show that overall measures of global health were largely unaffected. In fact, when compared to the published EORTC QLC published data for all cancer patients, our cohort reported significantly higher levels of global health. However, physical, role, and social functioning were markedly impaired when compared to healthy individuals. Until widespread vaccination and subsequent immunity is reached, and given that periodic “lockdowns” are likely to continue on a local and national level, there is a pressing need to fully understand the impact of the Covid-19 pandemic on quality of life in skin cancer patients in order to plan and provide psychosocial support and ensure that engagement with cancer care services is maintained in order to enable early detection of cancer progression and/or recurrence.
